# Relationship between intracranial internal carotid artery calcification and enlarged cerebral perivascular space

**DOI:** 10.1007/s00234-017-1838-7

**Published:** 2017-05-13

**Authors:** Xiao-Xiao Tao, Ge-Fei Li, Yi-Lan Wu, Yi-Sheng Liu, Ying Zhao, Yan-Hui Shi, Mei-Ting Zhuang, Tian-Yu Hou, Rong Zhao, Feng-Di Liu, Xue-Mei Wang, Ying Shen, Guo-Hong Cui, Jing-Jing Su, Wei Chen, Xue-Mei Tang, Ji Sun, Jian-Ren Liu

**Affiliations:** 1 Department of Neurology, Shanghai Ninth People’s Hospital, Shanghai Jiao Tong University School of Medicine, 639 Zhizaoju Road, Shanghai, 200011 China; 2 Department of Neurology, The First People’s Hospital of Wenling, Wenling, 317500 China; 3 Clinical Research Center, Shanghai Jiao Tong University School of Medicine, Shanghai, 200025 People’s Republic of China

**Keywords:** Intracranial internal carotid artery calcification, Cerebral enlarged perivascular space, PVS, Small vessel disease, Cerebral magnetic resonance imaging, MRI

## Abstract

**Purpose:**

The association between intracranial internal carotid artery (IICA) calcification and lacunes, white matter hyperintensity (WMH), and cerebral microbleeds (CMBs) has been well researched. However, enlarged cerebral perivascular space (PVS) has not yet been reported to correlate with intracranial internal carotid artery calcification. Therefore, the primary aim of this study was to investigate the relationship between IICA calcification and enlarged PVS.

**Methods:**

A total of 189 patients with ischemic stroke in the middle cerebral artery territory who presented within 7 days of ictus from 2012 to 2015 were enrolled respectively. All patients were required to have undergone head computed tomography, magnetic resonance imaging, susceptibility-weighted magnetic resonance imaging, magnetic resonance angiography, or computed tomography angiography. Clinical characteristics were recorded. IICA calcification and enlarged PVS were semi-quantitatively evaluated, and the presence of lacunes, WMH, and CMBs was recorded.

**Results:**

Of the 189 patients, 63.5% were male. Mean age of the patients was 68.6 ± 12.2 years. There were 104 patients with IICA calcification. Age, diabetes mellitus, lacunes, and white matter hyperintensity were significantly associated with IICA calcification (*P* < 0.05). Multivariate logistic regression analysis showed that age, diabetes mellitus, and lacunes were independent predictors of IICA calcification (*P* < 0.05). A lower risk of IICA calcification was found in patients with a higher enlarged PVS score (*P* = 0.004).

**Conclusion:**

Higher enlarged PVS scores were associated with a lesser degree of IICA calcification. There appears to be a relationship between reduced risk of IICA calcification and enlarged PVS.

## Introduction

Intracranial internal carotid artery (IICA) calcification is the most common type of intracranial arterial calcification [1, 2]. With the use of the Woodcock Visual Scoring system [3] and semiautomatic calculation of calcium score [4] as well as improvement in cranial computed tomography (CT) and computed tomography angiography (CTA), calcification of the IICA has been a focus in many studies. Currently, the relationship between IICA calcification and carotid atherosclerosis, incidence of stroke, and etiology of stroke is still controversial, and the mechanism underlying IICA calcification is still poorly understood.

Perivascular spaces (PVS), which are also called Virchow-Robin spaces (VRS), are normal anatomic structures in the nervous system [5]. They are formed when perforating arteries and outflow veins take the pia mater with them when they dive deep into the brain. Studies have confirmed that the Virchow-Robin spaces of capillary beds are occluded, and the interstitial fluid is static in the central nervous system [6]. The enlarged PVS have been found to be closely related to some pathological processes of the brain such as the atrophy of brain parenchyma and alteration of vascular permeability [7, 8]. On the basis of microscopic structural difference in pathophysiology, enlarged PVS can be mainly divided into two types. One is the basal ganglia type, in which the lenticulostriate arteries project into the basal ganglia via the anterior perforating arterial branch. The enlarged PVS of basal ganglia type has been confirmed to be closely related to cerebral small vessel disease (SVD). Basal ganglia-enlarged PVS have been reported to be associated with older age, centrum semiovale-enlarged PVS, cerebral atrophy, and lacunar stroke subtype [9, 10]; basal ganglia-enlarged PVS have also been associated with cognitive impairment [11]. The other type of enlarged PVS is the cerebral hemisphere type, in which medullary arteries enter the convex gray matter of the cerebral hemisphere and then extend to the subcortical white matter (centrum semiovale); thus, it is also known as cerebral white matter type. Neuroimaging studies have revealed that enlarged PVS, together with status lacunaris, white matter signal hyperintensity, and cerebral microbleeds, have been found to be markers related to cerebral SVD [9, 12, 13]. These four markers have been integrated as a system that is used to evaluate SVD (overall SVD scoring system) [14], which have been widely applied in clinical practice for the systemic evaluation of cerebral SVD.

In recent years, arterial calcification has been regarded as a discernible, highly specific marker of atherosclerosis in imaging. Coronary artery calcification has been confirmed to be related to coronary atherosclerotic plaques and can be used to predict the occurrence of coronary arterial events such as myocardial infarction and angina [15, 16]. There is evidence showing that the incidence of intracranial carotid calcification in stroke patients is significantly higher than in non-stroke subjects [2].

Many studies have shown that intracranial carotid calcification is closely related to status lacunaris, white matter signal hyperintensity, and microbleeds [17–21]. However, little is known about the relationship between intracranial carotid calcification and enlarged PVS as a kind of marker of cerebral SVD. Therefore, the primary aim of this study was to investigate the relationship between intracranial carotid calcification and enlarged PVS.

## Materials and method

### Patients

This was a retrospective study. The study included 189 patients diagnosed with ischemic stroke due to obstruction of the middle cerebral artery (MCA) at the Department of Neurology and Jiuyuan Municipal Stroke Center of Shanghai Ninth People’s Hospital, Shanghai Jiao Tong University School of Medicine between June 2012 and December 2015.

### Patient recruitment

The inclusion criteria were the following: (1) received treatments in the Department of Neurology of Shanghai Ninth People’s Hospital, Shanghai Jiao Tong University School of Medicine between June 2012 and December 2015; (2) had a first ischemic stroke, older than 40 years, unilateral obstruction of the MCA, and no hemorrhagic transformation (cranial CT showed hyperintensity at infarct area or cranial susceptibility-weighted magnetic resonance imaging (SWI) showed heterogeneous intensity); (3) underwent cranial plain MRI, cranial SWI, cranial plain CT, cranial magnetic resonance angiography (MRA), or cranial CT angiography. The interval between cranial plain MRI and cranial CT was no longer than 6 months; and (4) the images obtained were clear.

The exclusion criteria were the following: (1) evident malacia lesions in brain parenchyma, old infarction, hemorrhage, or tumor; (2) severe brain atrophy or hydrocephalus; (3) received thrombolytic therapy or revascularization (including arterial embolectomy and arterial stenting) before admission; (4) obvious artifacts in the images due to some reasons (such as denture); and (5) MRI parameters were adjusted due to special requirements.

### Clinical information at baseline

Age, gender, hypertension, diabetes mellitus (DM), hyperlipidemia, atrial fibrillation, coronary heart disease, and smoking status were recorded as risk factors of cerebrovascular diseases. Hypertension was defined as mean systolic blood pressure (SBP) >140 mmHg and/or mean diastolic blood pressure (DBP) >90 mmHg at two consecutive non-invasive measurements with an interval of at least 15 min, or a medical history of oral medication with blood pressure lowering drugs (such as diuretics, calcium channel blockers, angiotensin-converting enzyme inhibitors, etc.). Diabetes mellitus was defined as fasting plasma glucose >7.9 mmol/L, plasma glucose at any time of >11.0 mmol/L, or a history of oral medication of glucose lowering drugs. Hyperlipidemia was defined as low-density lipoprotein >160 mg/dL, total cholesterol of >240 mg/dL, triglycerides of >150 mg/dL, or a history of oral medication with lipid-lowering drugs. Coronary heart disease was defined as the presence of clinical manifestations of myocardial infarction, angina, or ischemic heart failure as well as coronary atherosclerosis. Smoking was defined as smoking >10 cigarettes per day or smoking for >1 year.

### Neuroimaging findings

All the patients underwent MRI scanning with a 3.0T MR scanner (Siemens, Germany), and images were obtained after axial scanning.

Cranial CT was performed with a 64-slice dual-source spiral CT (Siemens, Germany) (slice thickness 5 or 10 mm; FOV: 512 × 512).

Cranial MRI was performed with the following parameters: slice thickness: 5 mm; slice interval: 6.5 mm; T1WI sequence: TR, 1800 ms; TE, 8.8 ms, FOV, 640 × 576; T2WI sequence: TR, 4500 ms; TE, 106 ms; FOV, 640 × 576; FLAIR sequence: TR, 7000 ms; TE, 89 ms; FOV, 512 × 464; DWI sequence: TR, 4000 ms; TE: 100 ms; FOV, 288 × 324.

Susceptibility-weighted magnetic resonance imaging was performed with high-resolution, three-dimensional gradient-echo (3D-GE) sequence as follows: magnitude image (Mag): TR, 28.0 ms; TE, 20.0 ms; slice thickness, 1.5 mm; FOV, 640 × 520; phase image (Pha): TR, 28.0 ms; TE, 20.0 ms; slice thickness, 1.5 mm; FOV, 640 × 520; minimum intensity projection (mIP): TR, 28.0 ms; TE, 20.0 ms; slice thickness, 12.0 mm; FOV, 640 × 520; SWI image: TR, 28.0 ms; TE, 20.0 ms; slice thickness, 1.5 mm; FOV, 640 × 520.

### Neuroimaging analysis

The images on the PACS screen were evaluated by a neurologist and radiologist who were blind to the clinical characteristics and prognosis of these patients and received standardized training. The same template was used for determination of location, and degree of calcification, white matter hypersensitivity, status lacunaris, and microbleed were evaluated. Cerebral magnetic resonance angiography was performed with the following parameters: TR, 22 ms; TE, 3.6 ms; flip angle: 20o; slice thickness, 1.5 mm; slice interval, 1.8 mm; FOV, 640 × 580. Computed tomography angiography was performed with a 64-slice dual-source spiral CT (Siemens, Germany). Gadolinium solution was injected at 4.5 mL/s. The slice thickness was 1 mm; the FOV was 512 × 512.

### Degree of calcification

**Fig. 1:**
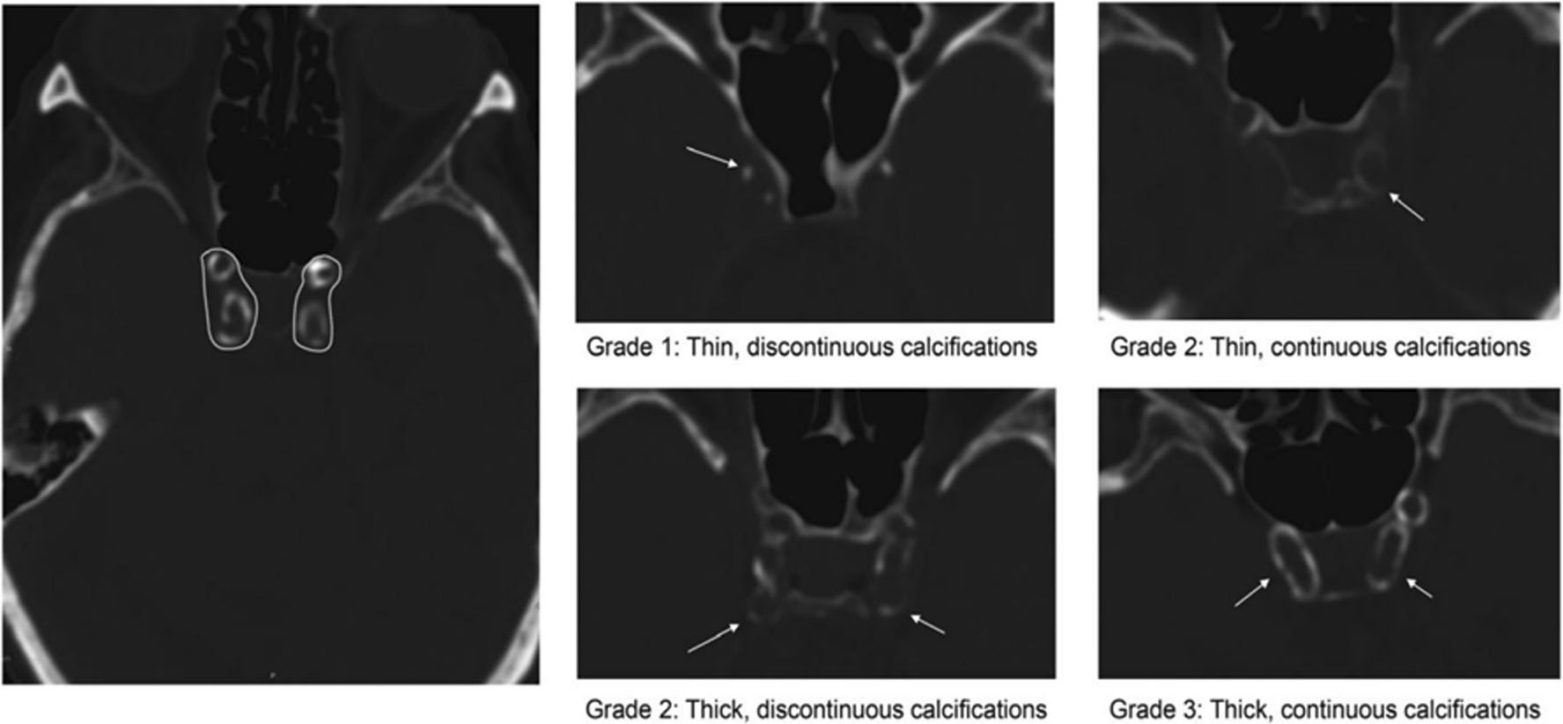
The area of interest for evaluating the degree of intracranial internal carotid artery calcification and examples for three grades

The bilateral intracranial carotid siphon was used as region of interest for the evaluation of degree of calcification. The consistent window (length 800 HU; width 2000 HU) was used in cranial plain CT, and the bilateral intracranial carotid siphon was evaluated with the Woodcock assessment system [3], with the highest degree recorded. Calcification was graded as 1, mild (thin and discontinuous calcification); 2, intermediate (thin and continuous calcification), thick or discontinuous calcification; 3, severe (thick and continuous calcification) (Fig. 1).

A. Semi-quantitative assessment of region of interest of the intracranial carotid artery: From petrous segment to internal carotid artery, bifurcation was performed.
B. Evaluation of calcification of the intracranial carotid artery was based on the following classification: grade 1 (thin and discontinuous calcification), grade 2 (thin and continuous calcification, or thick and discontinuous calcification), grade 3 (thick and continuous calcification).

#### Semi-quantitative assessment of perivascular space

**Fig. 2:**
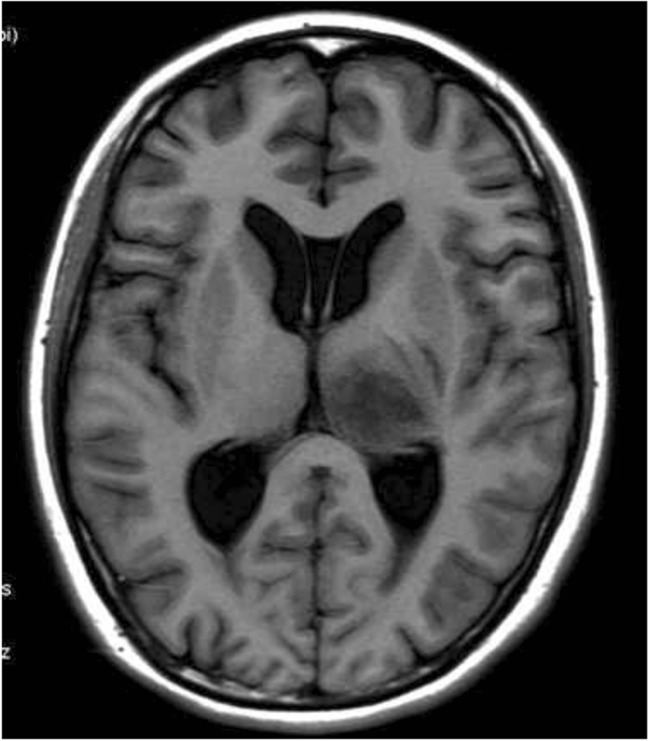
Axial cranial MRI localization slice at the level of the basal ganglia

**Fig. 3:**
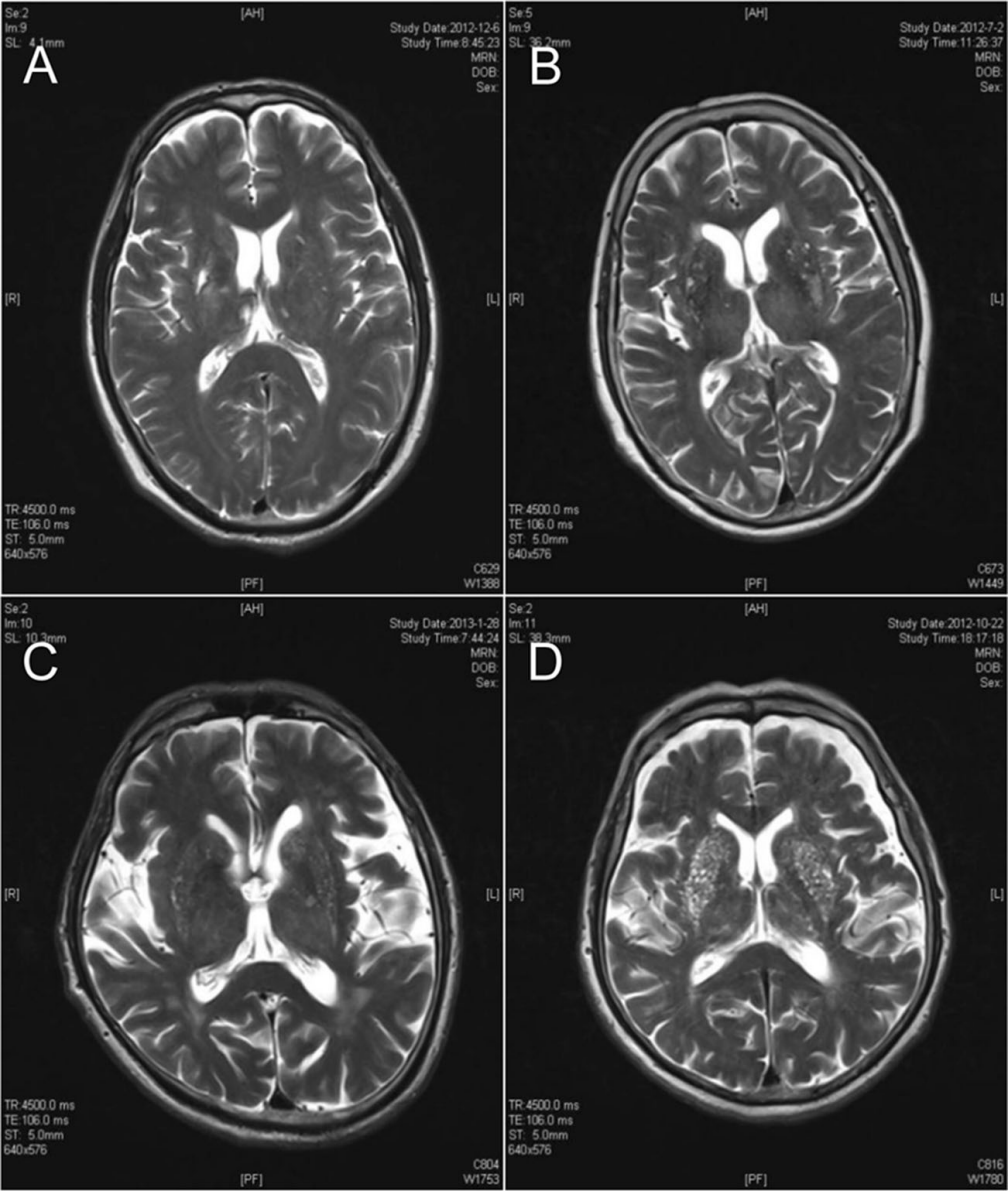
Example of different extent of enlarged perivascular space scoring from 1 to 4 at the level of the basal ganglia. **a** PVS for 1 point, **b** PVS for 2 points, **c** PVS for 3 points, **d** PVS for 4 points

The extent of PVS dilation was semi-quantitatively evaluated with the criteria developed by MacLullich et al. [22]: 0, no PVS; 1, <10 PVS; 2, 11–20 PVS; 3, 21–40 PVS; 4, >40 PVS. Counting of PVS was done in the hemisphere with the largest number. Figure 2 shows the location of the basal ganglia. Of note, macroscopic observation is more sensitive to hyperintensity than to hypointensity. In the present study, the PVS was counted on the T2WI sequence. Figure 3 shows the PVS of 1–4.

### Assessment of white matter hyperintensity

The criteria of Fazekas et al. [23] were employed to evaluate the deep white matter and white matter next to the lateral ventricle. Hyperintensity of white matter next to the lateral ventricle: 0, no lesion; 1, cat-like or pencil-like thin lesions; 2, lesion with smooth halo; 3, irregular hyperintensity next to the lateral ventricle extending to deep white matter. Hyperintensity of deep white matter: 0, no lesion; 1, spotty lesions; 2, lesions merged; 3, massive merged lesions.

**Fig. 4:**
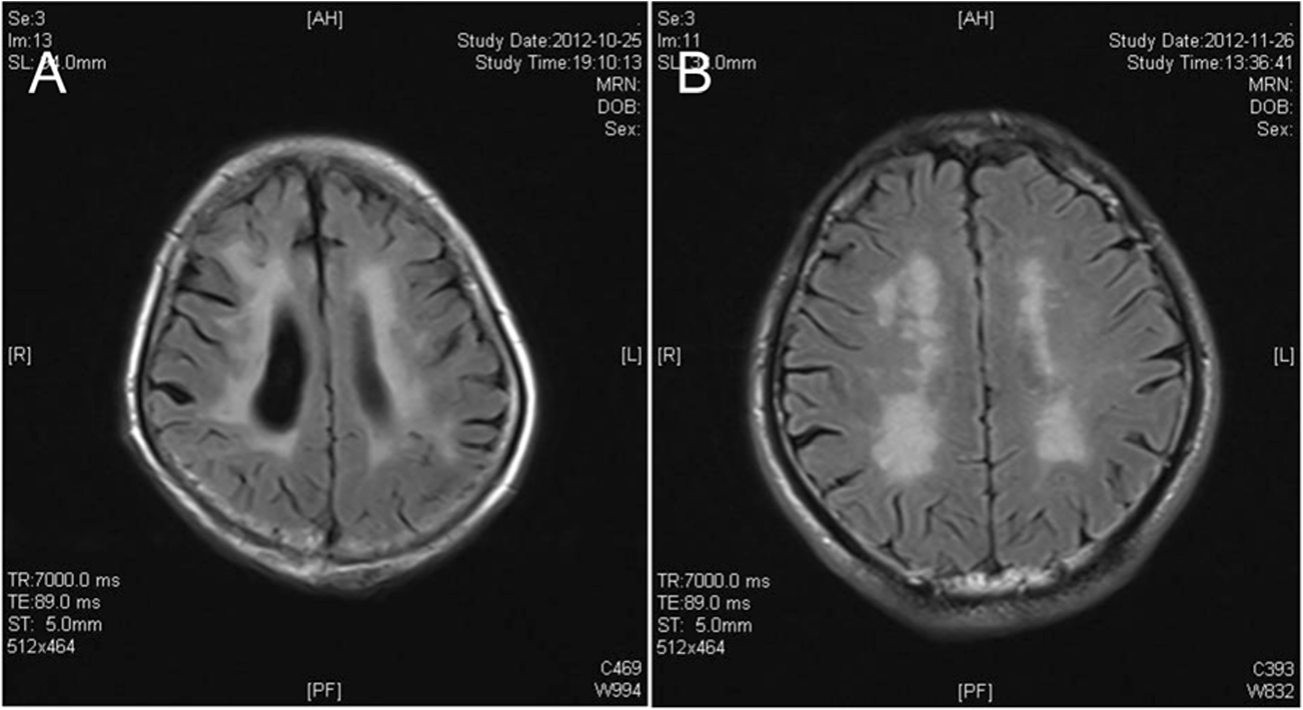
Example of periventricular and deep white matter hyperintensity. **a** High signal of lateral ventricle white matter with Fazekas 3 points (which elongate to deep white matter). **b** High signal in deep white matter with Fazekas 2 points (pathological changes of fusion)

The hyperintensity of white matter was defined as a score 3 for hyperintensity of white matter next to the lateral ventricle and/or score 2–3 of hyperintensity of deep white matter (Fig. 4). The presence of white matter hyperintensity was recorded. Of note, the white matter hyperintensity secondary to acute brain infarction was not included for evaluation.

### Assessment of status lacunaris

**Fig. 5:**
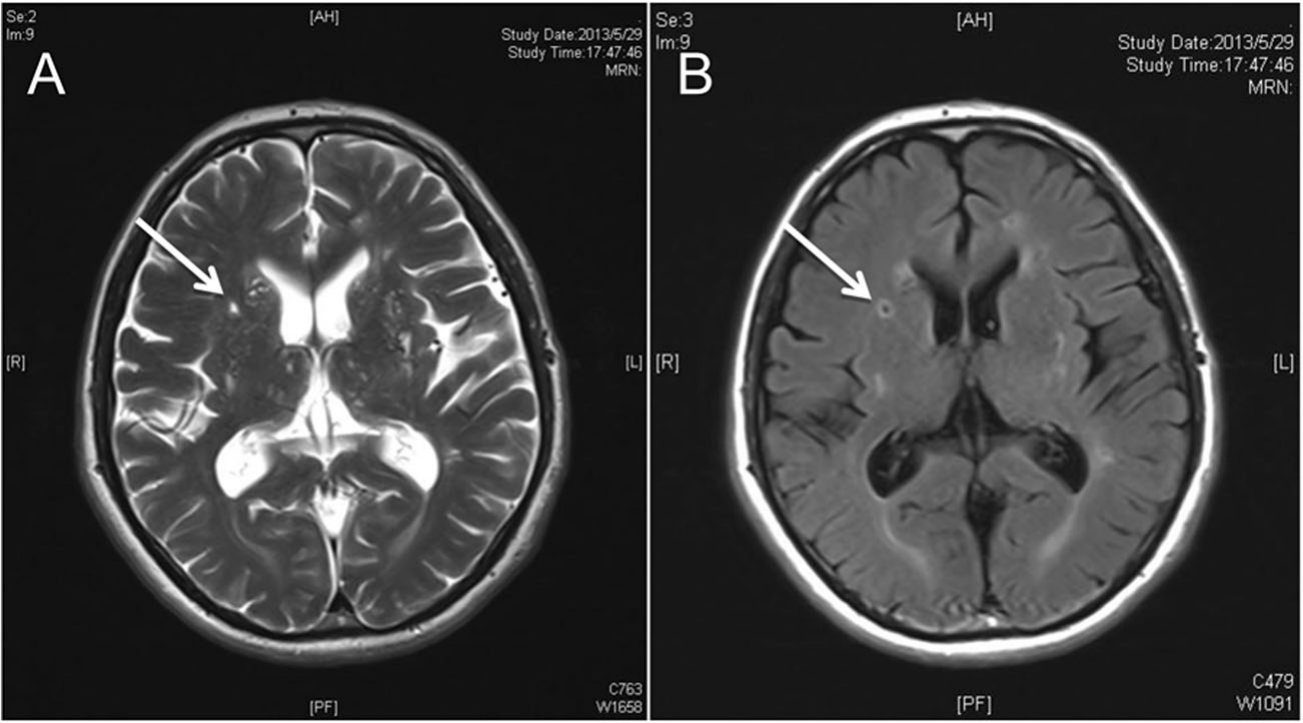
**a** Axial cranial T2 weighted MRI shows a lacunar infarct lesion of hyperintensity (*white arrow*). **b** Axial cranial FLAIR shows a lacunar infarct lesion with ring of hyperintensity and core of hypointensity (*white arrow*)

Status lacunaris, also known as lacunar infarction, and characterized by the round or oval lesions (3–20 mm in diameter) in the basal ganglia, internal capsule, centrum ovale, or brainstem which show similar intensity as cerebral spinal fluid on T2WI or T2 fluid-attenuated inversion recovery (FLAIR). Status lacunaris is hard to differentiate from enlarged PVS on T2WI and is mainly differentiated by FLAIR. Generally, on FLAIR, the lesions of lacunar infarction show hyperintensity at the periphery and hypointensity at the center; meanwhile, they have no hyperintensity on DWI [24] (Fig. 5). In this study, only the number of lacunar infarction lesions at the basal ganglia was counted.

### Evaluation of microbleeds

**Fig. 6:**
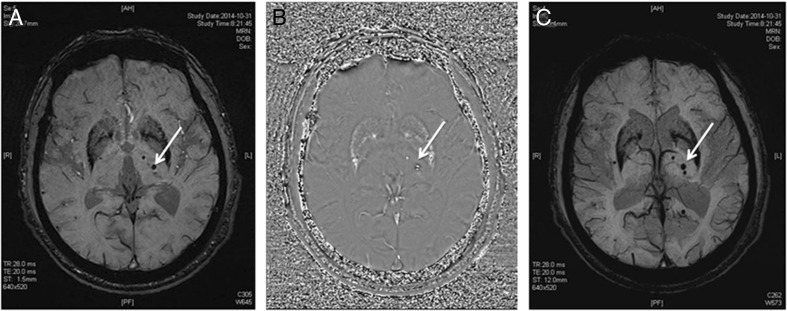
Different signals of cerebral microbleeds (CMBs) on sequences of SWI Phase and mIP. **a** SWI sequence: 2 lesions of microbleed on the left basal ganglia which are expressed as low signal (*white arrow*). **b** Phase sequence: 2 microbleed lesions on the left basal ganglia which are expressed as high signals (*white arrow*). **c** mIP sequence: 2 microbleed lesions on the left basal ganglia which are expressed as low signals (*white arrow*)

Microbleeds are defined as round or oval lesions (2–10 mm in diameter) with hypointensity on T2WI or SWI. Generally, they are not observable on cranial CT, FLAIR, T1WI, or T2WI. These lesions may be present in the cerebellum, brainstem, basal ganglia, white matter, and cortex–subcortex junction (21). Microbleeds are defined as one or more microhemorragic lesions in the brain parenchyma (Fig. 6). The presence of microbleeds was recorded. Microbleed lesions are also common in vascular amyloidosis (43) which is also a SVD. Thus, vascular amyloidosis was not excluded.

### TOAST subtyping

According to the subtyping criteria of the Trial of Org 10172 in Acute Stroke Treatment (TOAST) [19], the etiology of stroke was subtyped in each patient as follows: large artery atherosclerosis (LAA), small vessel occlusion (SVO), cardioembolism (CE), stroke of other determined etiology (SOE), and stroke of undetermined etiology (SUE).

### Statistical analysis

Mean and standard deviation were computed for age, and frequency and percentage were calculated for all categorical variables. To compare the difference between patients with and without IICA calcification, chi-square test or Fisher’s exact test for categorical variables and independent *t* test for age was used. Multivariate binary logistic regression analysis was implemented to determine the risk factors of IICA calcification based on the results of univariate analysis. Meanwhile, ordinal logistic regression was carried to examine the risk factors of severity of IICA calcification. Significance level was set at 0.05 for all analyses. All data were analyzed with PASW 22 (IBM Corp., Armonk, NY, USA).

## Results

Table 1 shows the characteristics of the patients with and without IICA calcification. There were some similarities between the two groups such as gender, smoking, hypertension, cardiovascular disease, TOAST classification, and cerebral microbleeds, but patients with IICA calcification tended to be older (72.9 vs. 63.4 years, *P* < 0.001), more prone to diabetes (39.4 vs. 17.6%, *P* = 0.001), and having 0-point enlarged PVS score (31.7 vs. 16.5%, *P* = 0.047), lacunes (68.3 vs. 36.5%, *P* < 0.001), and white matter hyperintensity (36.5 vs. 16.5%, *P* = 0.002). The final model revealed that advanced age (OR = 1.08, 95% CI 1.05–1.12, *P* < 0.001) was related to higher risk of IICA calcification. In addition, patients with diabetes or lacunes had about threefold (OR = 3.03, 95% CI 1.37–6.71, *P* = 0.006) or fourfold (OR = 4.24, 95% CI 2.04–8.80, *P* < 0.001) higher risk of IICA calcification compared with those without diabetes or lacunes. Conversely, a lower risk of IICA calcification was found in patients with 1-point (OR = 0.37, 95% CI 0.05–0.91, *P* = 0.030) or higher enlarged PVS score (OR = 0.17, 95% CI 0.06–0.48, *P* < 0.001) (Table 2).

**Table 1:** Clinical characteristics of 189 patients

	Intracranial internal carotid artery calcification		
	No (*n* = 85)	Yes (*n* = 104)	*P*
Age, years	63.4 ± 11.9	72.9 ± 10.8	*<0.001*
Males	58 (68.2)	62 (59.6)	0.221
Smoking	38 (44.7)	37 (35.6)	0.202
Hypertension	58 (68.2)	82 (78.8)	0.098
Diabetes	15 (17.6)	41 (39.4)	*0.001*
Hyperlipidemia	7 (8.2)	3 (2.9)	0.102
Cardiovascular disease	6 (7.1)	16 (15.4)	0.076
TOAST			0.506
Large artery atherosclerosis	14 (16.5)	20 (19.2)	
Small vessel occlusion	42 (49.4)	51 (49)	
Cardioembolism	3 (3.5)	8 (7.7)	
Undetermined etiology	26 (30.6)	25 (24)	
Enlarged PVS score			*0.047*
0	14 (16.5)	33 (31.7)	
1	44 (51.8)	41 (39.4)	
2–4	27 (31.7)	30 (28.9)	
Scanning slice thickness, mm			*0.002*
5	27 (31.8)	56 (53.8)	
10	58 (68.2)	48 (46.2)	
Lacunes	31 (36.5)	71 (68.3)	*<0.001*
Cerebral microbleeds	25 (29.4)	38 (36.5)	0.301
White matter hyperintensity	14 (16.5)	38 (36.5)	*0.002*

Data on age are presented as mean ± standard deviation and others are expressed as number (%).

Italicized values indicate statistically significant *P* < 0.05

*PVS* perivascular space, *TOAST* Trial of Org 10172 in Acute Stroke Treatment

**Table 2:** Risk factors for intracranial internal carotid artery calcification (*n* = 189)

	Odds ratio (95% confidence interval)	*P*
Age, years	1.08 (1.05, 1.12)	*<0.001*
Diabetes		
No	Reference	
Yes	3.03 (1.37, 6.71)	*0.006*
Score of enlarged perivascular space		
0	Reference	
1	0.37 (0.15, 0.91)	*0.030*
2–4	0.17 (0.06, 0.48)	*<0.001*
Lacunes		
No	Reference	
Yes	4.24 (2.04, 8.80)	*<0.001*

Forward logistic regression was performed.

Italicized values indicate statistically significant, *P* < 0.05.

Table 3 summarizes risk factors of severity of IICA calcification. In univariate analysis, age was found to be related to IICA calcification deterioration, and male gender was related to a minor severity; however, only the effect of age was significant in the multivariate analysis (OR = 1.04, 95% CI 1.01–1.08, *P* = 0.021).

**Table 3:** Risk factors for severity of intracranial internal carotid artery calcification in 104 patients

	Univariate		Multivariable	
	OR (95% CI)	*P*	OR (95% CI)	*P*
Age, years	1.06 (1.02, 1.1)	*0.003*	1.04 (1.01, 1.08)	*0.021*
Males	0.34 (0.16, 0.75)	*0.007*	0.46 (0.2, 1.03)	0.060
Smoking	0.57 (0.26, 1.24)	0.158		
Hypertension	0.81 (0.33, 1.99)	0.653		
Diabetes	0.94 (0.44, 1.97)	0.861		
Hyperlipidemia	8.69 (0.75, 100.92)	0.084		
Cardiovascular disease	1.47 (0.53, 4.07)	0.454		
TOAST				
Large artery atherosclerosis	Reference			
Small vessel occlusion	0.85 (0.41, 0.57)	0.665		
Cardioembolism	1.98 (0.57, 0.15)	0.281		
Undetermined etiology	0.8 (0.36, 0.56)	0.595		
Enlarged PVS score				
0	Reference			
1	0.55 (0.28, 0.95)	0.073		
2–4	0.73 (0.36, 0.68)	0.382		
Lacunes	0.7 (0.32, 1.54)	0.373		
Cerebral microbleeds	1.14 (0.53, 2.42)	0.744		
White matter hyperintensity	1.15 (0.54, 2.46)	0.718		

Italicized values indicate statistically significant *P* < 0.05

*CI* confidence interval, *OR* odds ratio, *PVS* perivascular space, *TOAST* Trial of Org 10,172 in Acute Stroke Treatment

## Discussion

In this study, which is the first to investigate the association between IICA calcification and enlarged cerebral PVS, it was found that enlarged PVS were associated with a lower risk of IICA calcification. Multivariate logistic regression analysis showed that age was an independent risk factor for increased severity of IICA calcification. This finding is consistent with a previously reported finding that calcification volume in IICA calcification is positively associated with age [25].

In previous studies on coronary arterial calcification and calcification of common carotid bifurcation, the incidence of calcification was higher and the calcification volume was larger in males [26–28]. This may be related to the higher prevalence of smoking, drinking, and hyperlipidemia in males than in females as these are risk factors for cerebrovascular diseases. Our results showed that male gender was negatively related to calcification, and there was significant difference in gender among patients with different degrees of calcification, and more patients with mild calcification were males. However, the sample size was relatively small, and ischemic stroke patients were included. Thus, our findings require further confirmation.

There is evidence showing that DM is not only an independent risk factor for intracranial carotid calcification [29, 30] but is closely related to calcification of the extracranial carotid artery, coronary artery, and aortic arch [31, 32]. Yilmaz et al. [32] also found that DM was independently related to the calcification loading of intracranial carotid calcification. In this study, DM, age, and status lacunaris as risk factors of cerebrovascular diseases were included in multivariate logistic regression analysis, and the results also showed DM was independently associated with intracranial carotid calcification (*P* < 0.05). Diabetes mellitus may cause high inflammatory state, abnormal lipid metabolism, and abnormal activity of the fibrinolytic system and has been regarded as an important factor involved in the initiation and progression of atherosclerosis [33]. Our findings may be helpful for the elucidation of the relationship between intracranial carotid calcification and atherosclerosis and provide evidence for future studies in this field.

Enlarged PVS (EPVS) were identified on T2WI and MPRAGE T1 and defined as round, ovoid, or linear structures with CSF-like signal, no larger than 3 mm in diameter and located in territories supplied by perforating arteries. EPVS in both basal ganglia and centrum semiovale regions were rated according to a previously published 4-point semi-quantitative score [9, 34]. We assessed EPVS in the basal ganglia and centrum semiovale separately because it was possible that due to their different locations and features, they could have distinct pathophysiology. EPVS in both the basal ganglia and centrum semiovale were coded with the following scale applied to standard axial images: 0 indicating no EPVS, 1 indicating 10 EPVS, 2 indicating 11 to 20 EPVS, 3 indicating 21 to 40 EPVS, and 4 indicating more than 40 EPVS. The numbers referred to PVS on one side of the brain; the higher score was used if there was asymmetry between the sides and EPVS were counted in the slice with the highest number.

Our results showed the grade of enlarged PVS was similar between patients with and without calcification and in those with different degrees of calcification. However, multivariate logistic regression analysis showed enlarged PVS was associated with a reduced risk for IICA calcification (*P* = 0.004), i.e., the higher the enlarged PVS score, the milder the calcification. The non-significant difference in the score of enlarged PVS might be ascribed to small number of patients with enlarged PVS of grade 3–4 and the abnormal distribution of enlarged PVS of different grades.

In the present cross-sectional study, it was not possible to clarify the cause–effect relationship between increased enlarged PVSs and reduced risk of intracranial internal carotid artery calcification. The causal relationship should be confirmed in prospective studies with long-term follow-up. Our findings suggest that enlarged PVS are different from other cerebral SVDs, such as status lacunaris, white matter signal hyperintensity, and microbleeds, that are positively related to cerebrovascular calcification [17–21] and that the pathogenic mechanism of enlarged PVS is different from that of cerebrovascular calcification. This provides evidence for the need to further explore the mechanism underlying the pathogenesis of enlarged PVS.

It should also be noted that basal ganglia-enlarged PVS have been associated with the lacunar stroke subtype [9, 10], deep and periventricular white matter hyperintensity [9], and cognitive impairment. On the other hand, centrum semiovale-enlarged PVS were not found to be associated with any particular ischemic stroke subtype, deep or periventricular white matter hyperintensity [9], or cognitive impairment [35]. That is why we only analyzed the association between basal ganglia enlarged PVS and intracranial internal carotid artery calcification.

Our study had several limitations. This was a retrospective study. Also, the sample size was small due to time and recruitment limitations. Thus, all the risk factors related to intracranial carotid calcification were not covered in this study, and future prospective studies with large sample size are required to confirm our findings. In addition, there might be selection bias in the patients’ recruitment because patients with severe stroke are unable to undergo cranial MRI and other imaging examinations. Thus, the results obtained in this study might be biased.

## Conclusion

Our results showed basal ganglia-enlarged PVS are associated with a reduced risk of IICA calcification, i.e., the higher the grade of PVS, the milder the calcification. This is the first study to investigate the relationship between PVS dilation and IICA calcification. In addition, our results also showed age, DM, and status lacunaris were independent predictors of IICA calcification, which is consistent with previous reports.

## Abbreviations

IICA: Intracranial internal carotid artery
PVS: Perivascular spaces
WMH: White matter hyperintensity
CMB: Cerebral microbleeds
SVD: Small vessel disease
MRI: Magnetic resonance imaging
SWI: Susceptibility-weighted magnetic resonance images
MRA: Magnetic resonance angiography
CTA: Computed tomography angiography
T1WI: T1-weighted imaging
T2WI: T2-weighted imaging
DWI: Diffusion-weighted imaging
FLAIR: Fluid-attenuated inversion recovery
mIP: Minimum intensity projection
LAA: Large artery atherosclerosis
SVO: Small vessel occlusion
CE: Cardioembolism
SOE: Stroke of other determined etiology
SUE: Stroke of undetermined etiology
